# STX‐0119, a novel STAT3 dimerization inhibitor, prevents fibrotic gene expression in a mouse model of kidney fibrosis by regulating Cxcr4 and Ccr1 expression

**DOI:** 10.14814/phy2.14627

**Published:** 2020-10-28

**Authors:** Kouki Makitani, Naohisa Ogo, Akira Asai

**Affiliations:** ^1^ Center for Drug Discovery Graduate School of Pharmaceutical Sciences University of Shizuoka Shizuoka Japan

**Keywords:** Ccr1, Cxcr4, kidney fibrosis, STAT3 inhibitor

## Abstract

Kidney fibrosis is a histological hallmark of chronic kidney disease (CKD) and is believed to be involved in the progression of CKD. Therefore, inhibition of kidney fibrosis is a potential strategy for slowing CKD progression. Signal transducer and activator of transcription 3 (STAT3) is a transcription factor that is activated by interleukin‐6 and is reported to be involved in fibrosis. Previously, S3I‐201, an inhibitor of STAT3 phosphorylation, was shown to inhibit renal fibrosis in a mouse model, but its mechanism was not clarified completely. In this study, we investigated whether STX‐0119, a new inhibitor of STAT3 dimerization, suppressed kidney fibrotic gene expression using a mouse model of kidney fibrosis and examined the underlying mechanisms. Kidney fibrosis was induced by unilateral ureteral obstruction (UUO), which was accompanied by upregulation of STAT3 target genes. STX‐0119 administration suppressed the expression of fibrotic genes in UUO kidneys without affecting STAT3 phosphorylation. STX‐0119 decreased Cxcr4 mRNA in cultured rat kidney fibroblasts and Ccr1 mRNA in blood cells from UUO mice, both of which are reported to be involved in the progression of kidney fibrosis. These results suggest that STX‐0119 inhibits fibrotic gene expression in kidney by suppressing Cxcr4 and Ccr1 expression. This is the first report to indicate a part of the mechanism of the antifibrotic effects of a STAT3 inhibitor and suggests that STX‐0119 may be a lead compound for the treatment of kidney fibrosis.

## Graphical abstract

A STAT3 dimerization inhibitor STX‐0119 suppresses fibrotic gene expression in a mouse UUO model by regulating Cxcr4 and Ccr1 expression

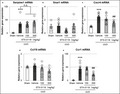

## 1 INTRODUCTION

Chronic kidney disease (CKD) is a progressive condition and the number of CKD patients worldwide is increasing each year. Patients with CKD require treatment by kidney transplantation or dialysis, but there are problems such as a lack of kidney donors, posttransplant rejection, and dialysis complications. As a result, poor quality of life in patients with CKD and rising medical expenses associated with the increase in CKD patients have become major social issues.

Kidney fibrosis is a histological hallmark of CKD (Risdon et al., (1968); Nath, 1992). Fibrosis is supposed to be a common pathway to end‐stage renal disease (ESRD), but the mechanism remains unclear. Kidney fibrosis is induced by an increase in extracellular matrix (ECM) protein production (Bülow & Boor, 2019), a decrease in matrix degradation (Ha and Lee, (2003); Han et al., 2006), inflammatory cell infiltration (Black et al., 2019; Lv et al., 2018), and transformation of resident cells (Chevalier et al., (2009); Vega et al., 2016; Qi et al., 2006). Many researchers have tried to address these situations. A representative approach is antitransforming growth factor‐β (TGF‐β) therapy (Gagliardini & Benigni, 2006; Isaka, 2018; Meng et al., 2016). TGF‐β is a multifunctional cytokine that is reported to regulate ECM accumulation, cell proliferation, and the synthesis of individual matrix components (Ihn, 2002; López‐Hernández & López‐Novoa, 2012; Sutariya et al., 2016). However, anti‐TGF‐β therapy has not shown efficacy in patients with diabetic kidney disease or focal segmental glomerulosclerosis (FSGS) (Trachtman et al., 2011; Vincenti et al., 2017; Voelker et al., 2017). Therefore, delayed CKD progression by antifibrotic therapy has not been achieved, and researchers are trying to overcome kidney fibrosis in the belief that antifibrotic treatment will help to decrease the number of patients progressing to ESRD.

Signal transducer and activator of transcription 3 (STAT3) is a transcription factor activated by interleukin‐6 (IL‐6) or TGF‐β 1 (Shizuo, 1997; Tang et al., 2017) and is expressed in various cell types, such as leukocytes and fibroblasts (Deenick et al., 2018; Kasembeli et al., 2018). Upon the activation of cytokine receptors, STAT3 is recruited and phosphorylated at a tyrosine residue adjacent to the SH2 domain by receptor‐type tyrosine kinases such as Janus‐activated kinases. Once phosphorylated, STAT3 forms a homodimer by protein–protein interactions. STAT3 dimerization induces the nuclear transport of STAT3, resulting in STAT3 binding to DNA and upregulating the expression of its target genes (Darnell, 1997; Heinrich et al., 1998; Levy & Darnell, 2002). STAT3 target genes encode various factors, including cytokines, growth factors, and ECM components, that contribute to tissue fibrosis (Ray et al., 2013; Hillmer et al., 2016). Hence, inhibition of STAT3 activation may delay the progression of kidney fibrosis.

STX‐0119 was identified by virtual screening as an inhibitor of STAT3 dimerization (Matsuno et al., 2010). STX‐0119 inhibits STAT3 dimerization without affecting its phosphorylation state. STX‐0119 has been shown to exert antitumor effects in vitro and in vivo*(*Ashizawa, 2011; Ashizawa et al., 2013; Ashizawa et al., 2014; Akiyama et al., 2017) and antifibrotic effects in a mouse model of liver fibrosis (Choi et al., 2019). In addition, S3I‐201, an inhibitor of STAT3 phosphorylation, has been shown to have antifibrotic effects in a unilateral ureteral obstruction (UUO) model by repressing STAT3 phosphorylation (Pang et al., 2010). However, no STAT3 dimerization inhibitor has been assessed for the possibility of antifibrotic effects in kidneys, and the detailed mechanisms of the antifibrotic effects of STAT3 inhibitors remain to be elucidated.

From these reports, we hypothesized that STX‐0119 would exert antifibrotic effects in the kidney, and examined its effects on kidney fibrotic gene expressions using a mouse model of kidney fibrosis. In addition, we analyzed the mechanism of STX‐0119 by focusing on resident fibroblasts and immune cells in the kidney.

## 2 MATERIALS AND METHODS

### 2.1 Cell culture

NRK‐49F and RAW264.7 cells were obtained from the American Type Culture Collection (Manassas, VA). Fresh media were replaced every 3 days, the cells were maintained at subconfluence for between 3 and 10 passages. NRK‐49F cells were grown in Dulbecco's modified Eagle's medium/F12 containing 5% heat‐inactivated fetal bovine serum and 1% streptomycin‐penicillin. RAW264.7 cells were grown in RPMI‐1640 medium containing 10% heat‐inactivated fetal bovine serum and 1% streptomycin‐penicillin. Both cell lines were maintained in an atmosphere of 5% CO2 and 95% air at 37°C in a humidified incubator. For experiments, the cells were collected by 0.25% trypsin treatment and seeded in 96‐well clear plates (Greiner Bio‐One, Tokyo, Japan) at density of 1.0 × 10^4^ cells/well. Recombinant rat IL‐6 protein, recombinant mouse IL‐6 protein, and recombinant TGFβ 1 were purchased from R&D systems (USA).

### 2.2 Animals

Male 8‐week‐old C57BL/6 mice (20‐25 g, Charles River Japan, Inc., Japan) were used in the in vivo study. Animals were housed five per case (W235 × D353 × H160 mm) under specific pathogen‐free condition. The mice were kept at 19–25°C in 30%–70% humidity under a 12‐hr light–dark cycle with ad libitum access to tap water and commercial chow (FR‐2; Funabashi Farm, Chiba, Japan). All animals received humane care that complied with the Japanese Pharmacological Society's “Guiding Principles for the Care and Use of Laboratory Animals.”

### 2.3 UUO surgery

The mice were grouped randomly just before the surgery. The mice were anesthetized by intraperitoneal administration of pentobarbital (60mg/kg), and incisions were made along the dorsal side. The left ureter was isolated from the surrounding tissues and subsequently double ligated. The partial ureter between the two ligations was cut by surgical scissors. The incisions were sutured with sterile threads. In order to relieve the pain, mice were treated with buprenorphine (0.1 mg/kg, subcutaneous administration) immediately after the surgery. Mice were put on heat pad and maintain body temperature at about 37℃ until awaking. We performed euthanasia by cutting abdominal aorta under the condition in which mice were anesthetized with isoflurane.

### 2.4 Preparation and administration of STX‐0119

Administration solution of STX‐0119 was prepared by suspending STX‐0119 in 0.5% methyl cellulose 400 solution (Wako, Japan). STX‐0119 was weighed using electronic weighing instrument and amorphized by a mortar. 0.5% methyl cellulose 400 solution was added to amorphized STX‐0119 for making the solutions of designated concentration. STX‐0119 and vehicle (0.5% methyl cellulose 400) solution were administrated by oral gavage (at volume of 5 ml/kg) 1h before the UUO surgery and once a daily for subsequent two days by the same method.

### 2.5 Extraction of mRNA from blood, kidney tissues, and cell culture and reverse transcription

Blood cells: mRNA from blood cells was extracted using a QIAamp RNA Blood Mini Kit (Qiagen, Hilden, Germany). cDNA was synthesized using a SuperScript™ VILO™ cDNA Synthesis Kit (Invitrogen, Carlsbad, CA). The kits were used according to the manufacturers’ protocols.

Kidney tissues: mRNA from kidney tissues was extracted using a Maxwell® RSC simplyRNA Tissue Kit (Promega, Madison, WI). The following experiments were conducted according to the same procedures for blood cells.

Cell culture: mRNA extraction and cDNA synthesis were performed using a SuperPrep® Cell Lysis & RT Kit for qPCR (Toyobo, Osaka, Japan) according to manufacturer's protocol.

### 2.6 Taqman real‐time quantitative RT‐PCR

Real‐time PCR was performed with TaqMan assay systems. Each TaqMan probe ID is shown in Table 1. Briefly, we made mixtures of 10X diluted reverse transcription products with TaqMan Gene Expression Master Mix (Applied Biosystems, Foster City, CA) containing each probe targeting the gene of interest. PCR reactions were performed under the following conditions: 50°C for 2 min, 95°C for 10 min, and 40 cycles of 95°C for 15 s and 60°C for 1 min. Relative gene expression was calculated by the standard curve method.

### 2.7 RT^2^ Profiler PCR Arrays

Synthesized cDNAs from each group (*n* = 4) were mixed in an equal amount to one sample for each group. Mixtures were loaded on an RT2 Profiler PCR Array Kit for Fibrosis, Inflammatory Cytokines and Receptors, or IL‐6/STAT3 Signaling Pathway according to the manufacturer's instructions (Qiagen). Fold change in mRNA expression was calculated by determining the ratio of mRNA levels to control or sham values using the ΔΔCt method. All data were normalized to an average of five housekeeping genes (Gusb, Hprt, Hsp90ab1, Gapdh, and Actb). The following PCR conditions were used: 10 min at 95°C, followed by 45 cycles of 15 s at 95°C and 60 s at 60°C.

### 2.8 AlphaLISA

Protein expression of total STAT3 and p‐STAT3 (Tyr705) was determined using an AlphaLISA SureFire Ultra Phospho‐STAT3 (Tyr705) Kit and AlphaLISA SureFire Ultra Total STAT3 Kit (PerkinElmer, Waltham, MA) according to the manufacturer's instructions. Kidney and cell lysates were prepared using the lysis buffer attached to these kits. Lysates of blood cells were prepared by the following method: whole blood was obtained from the abdominal vena cava of mice anesthetized with isoflurane using 1‐mL syringesand 25G heparin‐coated needles (Terumo, Tokyo, Japan), and hemolysis was achieved by RBC Lysis Buffer (BioLegend, San Diego, CA). Hemolytic samples were centrifuged and washed three times with phosphate‐buffered saline (Gibco, Grand Island, NY), and finally the washed pellets were lysed with a lysis buffer.

### 2.9 Enzyme‐linked immunosorbent assay (ELISA)

IL‐6 protein in mouse kidney was determined using an IL‐6 Mouse Uncoated ELISA Kit with Plates (Invitrogen) according to the manufacturer's protocol. Kidneys were lysed with RIPA buffer (Thermo Fisher Scientific, Waltham, MA), and protein concentrations were calculated by a BCA protein assay (Thermo Fisher Scientific).

### 2.10 Statistical analysis

Data are shown as the mean ± standard error of the mean and raw form (◇). One‐way analysis of variance, followed by Tukey's *post hoc* test, was used to compare multiple treatment groups. Statistical significance was set at *p*‐values <.05.

## 3 RESULTS

### 3.1 STAT3 is activated in UUO kidneys

**Figure 1:**
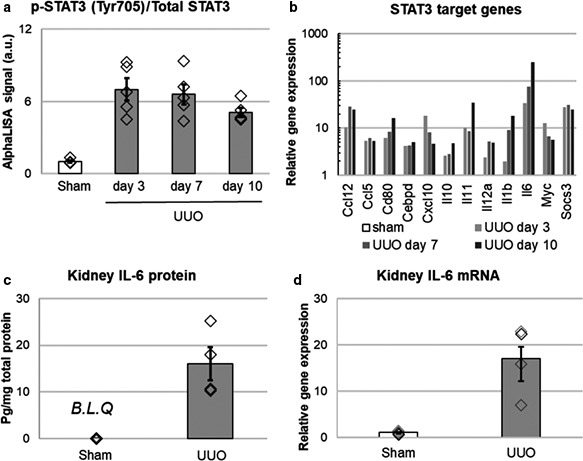
STAT3 activation in UUO mouse kidneys. (a) Phosphorylation state of STAT3 in UUO kidneys. Protein levels of p‐STAT3 and total STAT3 in kidney lysates were determined by AlphaLISA. *N* = 5 for each. (b) IL6/STAT3 signaling target genes in UUO kidney. Kidney mRNA was extracted and reverse transcribed. Expressions of mRNA was detected using IL6/STAT3 Signaling Pathway PCR Array (RT^2^Profiler^TM^ PCR Array). The values of each gene expressions were shown in semilogarithmic graph. Fold change in mRNA expression was calculated by determining the ratio of mRNA levels to sham values. (c, d) Expression of IL‐6 protein (c) and mRNA (d) in UUO kidneys (day3). *N* = 4 for IL‐6 protein experiment (c) and *N* = 5 for IL‐6 mRNA experiment (d). mRNA was detected by RT‐qPCR, and protein expression was detected by ELISA. B.L.Q: below the limit of quantification

Previously, western blot and immunohistochemistry analyses have indicated that STAT3 phosphorylation is increased in UUO mouse kidneys (Pang et al., 2010). In this study, we examined STAT3 phosphorylation by AlphaLISA, a more quantitative assay system. We detected the upregulation of phosphorylated p‐STAT3 (Tyr705)/total STAT3 level in UUO kidneys (Figure 1a); furthermore, the expression of STAT3 target genes was increased after UUO surgery (Figure 1b). We also investigated the expression of IL‐6, a major activator of STAT3. The expression of IL‐6 mRNA and protein was upregulated in UUO kidneys (Figure 1c‐d). These data indicate that STAT3 is activated in obstructed kidneys.

### 3.2 STX‐0119 inhibits fibrotic gene expression in UUO kidneys

**Figure 2:**
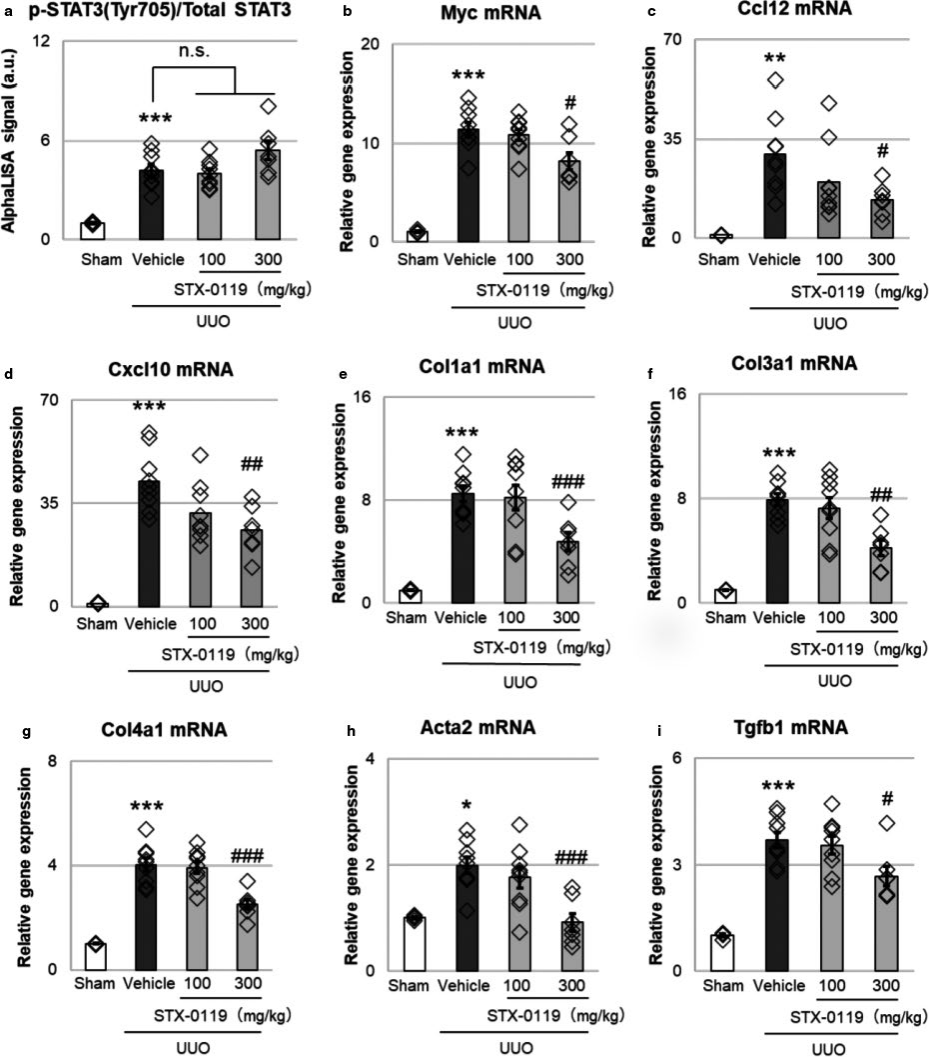
Effects of STX‐0119 on STAT3 activation and fibrotic genes in mouse UUO kidneys. STX‐0119 was administered once daily for 3 days. The mice were sacrificed at day 3 after UUO surgery. Each gene was normalized by Gapdh mRNA. (a) Effects of STX‐0119 on p‐STAT3/total STAT3 in mouse UUO kidneys. p‐STAT3 and STAT3 protein expression levels were evaluated by AlphaLISA. (b–d) Effects of STX‐0119 on STAT3 downstream genes (Myc [b], Ccl12 [c], and Cxcl10 [d]) in mouse UUO kidneys. (e–i) Effects of STX‐0119 on fibrotic genes in mouse UUO kidneys. *p < .05, **p < .01, ***p < .001 versus sham; #*p* < .05, ##*p* < .01, ###*p* < .001 versus UUO + Vehicle; one‐way analysis of variance + Tukey's test. n.s., not significant. *N* = 4 for sham group and *N* = 9 for other groups. These results were replicated by three times at independent experiments

Treatment with STX‐0119 (160 mg kg^‐1^ day^‐1^) inhibits the tumor growth of glioblastoma multiforme stem‐like cell lines transplanted in mice (Ashizawa, 2011). In this study, we selected 100 and 300 mg kg^‐1^ day^‐1^ STX‐0119 for in vivo experiments to inhibit STAT3 in the kidney. STX‐0119 did not affect STAT3 phosphorylation in UUO kidneys (Figure 2a); however, it decreased the expression of STAT3 target genes such as Myc, Ccl12, and Cxcl10 (Figure 2b–d). We examined the expression of Col1a1, Col3a1, Col4a1, and Acta2 as markers of kidney fibrosis, and Tgfb1, which is known to exacerbate kidney fibrosis (Sutariya et al., 2016). The oral administration of STX‐0119 significantly inhibited the expression of these genes (Figure 2e–i). These results suggest that STX‐0119 inhibits STAT3 activation without affecting STAT3 phosphorylation; furthermore, it prevents upregulation of fibrotic genes in obstructed kidneys.

### 3.3 STX‐0119 suppressed leukocyte marker expressions in UUO kidney

**Figure 3:**
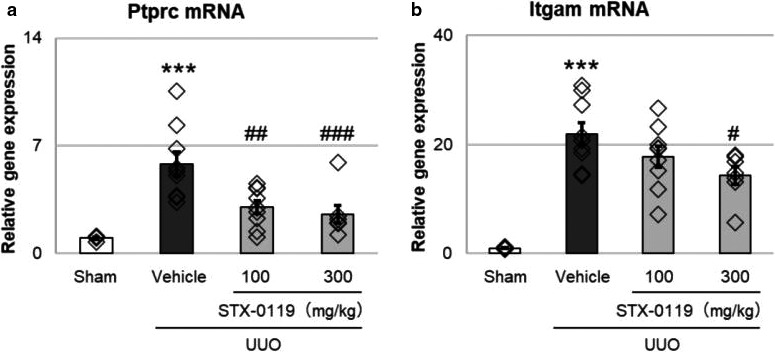
Effects of STX‐0119 on leukocyte markers in UUO kidneys. The mice were sacrificed at day 3 after UUO surgery, and mRNA was extracted and reverse transcribed. Gene expression of Ptprc (a) and Itgam (b) was detected by qPCR. Both genes were normalized by Gapdh mRNA. ***p < .001 versus sham; #*p* < .05, ##*p* < .01, ###*p* < .001, versus UUO + Vehicle; one‐way analysis of variance + Tukey's test. *N* = 4 for sham group and *N* = 9 for other groups

To explore the mechanism of action of STX‐0119, we focused on immune cell infiltration in the kidney. First, we examined the mRNA expression of Ptprc, a marker of leukocytes, and Itgam, a marker of myeloid cells, such as macrophages and monocytes. STX‐0119 administration decreased the gene expression of Ptprc and Itgam in UUO kidneys (Figure 3a, b). These results suggest that STX‐0119 suppressed the expression of immune cell markers in UUO kidneys and it can be speculated to inhibit the infiltration of immune cells into obstructed kidneys, and this effect may be involved in the antifibrotic effects of STX‐0119.

### 3.4 STAT3 activation upregulates the expression of various genes in vitro

**Figure 4:**
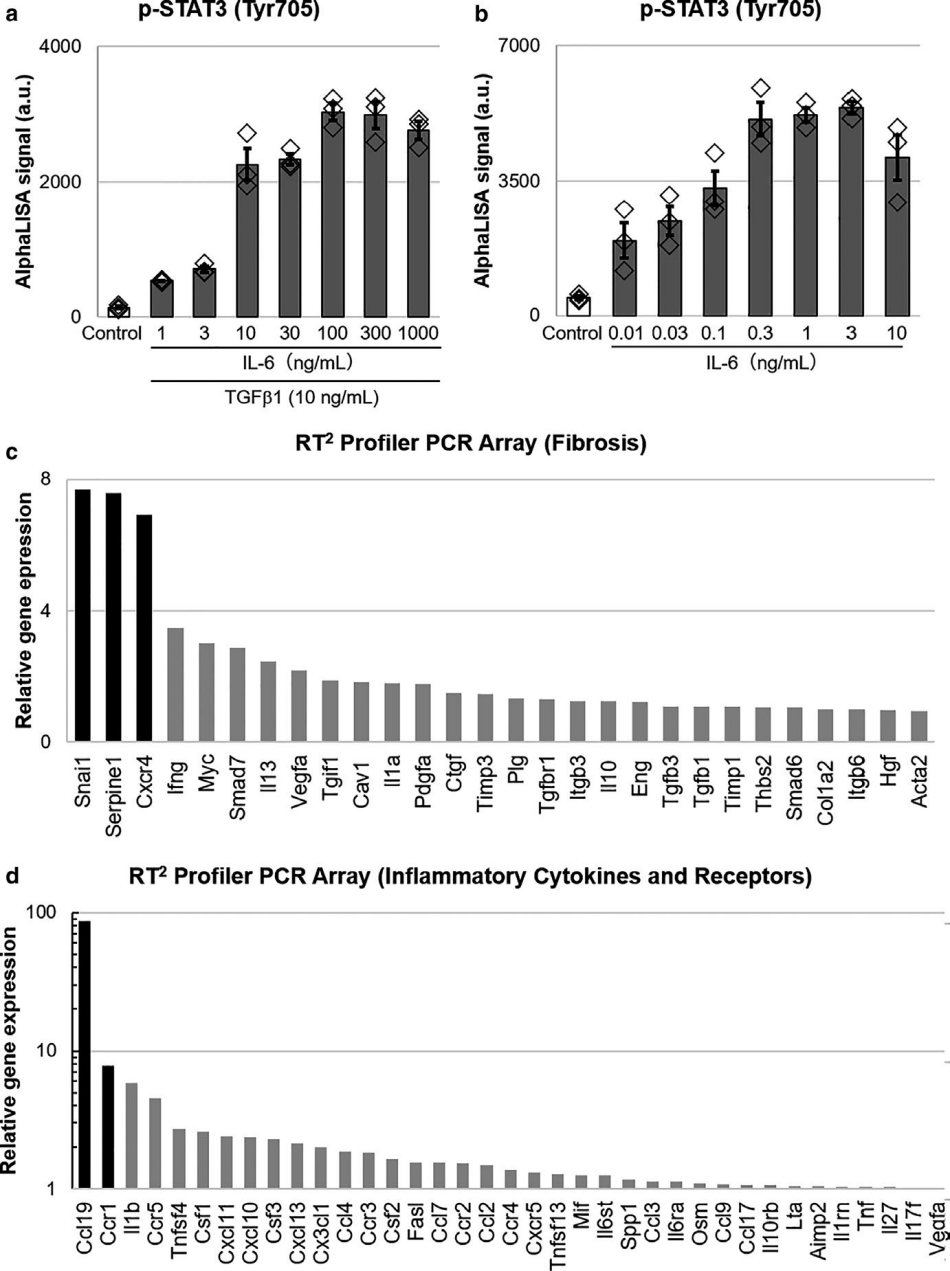
Relative gene expression of IL‐6‐ and TGFβ1‐treated NRK‐49F cells, and IL‐6‐treated RAW264.7 cells. (a,b) STAT3 phosphorylation in NRK‐49F cells (a) and RAW264.7 cells (b) were evaluated by AlphaLISA. The cells were treated with IL‐6 and TGF‐β 1 (a) or IL‐6 only (b) for 10min. *N* = 3. (c,d) Relative gene expression of IL‐6 (10 ng/ml)‐ and TGFβ 1 (10 ng/ml)‐treated NRK‐49F cells (c), and IL‐6 (0.1 ng/ml)‐treated RAW264.7 cells (d). The cells were treated for 1h and mRNA was extracted. Gene expression was detected using an RT^2^ Profiler PCR Array Kit. Data are presented as fold change to control, and only upregulated genes are shown

Because it was suggested that STX‐0119 inhibited the infiltration of immune cells into UUO kidneys, we examined which molecules are involved in the effects of STX‐0119 using RAW264.7 cells, a mouse macrophage cell line. In addition, we assumed that tubular interstitial cells were targets of STX‐0119 because STAT3 is expressed in fibroblasts. Therefore, we also tried to identify the key molecules using NRK‐49F cells, a rat kidney fibroblast cell line. We investigated which genes were upregulated by STAT3 activation in each cell type. STAT3 phosphorylation was increased drastically in NRK‐49F cells treated with IL‐6 (>3 ng/ml) and TGFβ 1 (10 ng/ml) (Figure 4a), and in RAW264.7 cells treated with IL‐6 (>0.03 ng/ml) (Figure 4b). Therefore, in order to activate stat3, we treated NRK‐49F cells with IL‐6 (10 ng/ml) and TGFβ 1 (10 ng/ml), and RAW264.7cells with IL‐6 (0.1 ng/ml) in the following experiments. To explore disease‐related genes, we used RT^2^ Profiler PCR Arrays, by which we could analyze the expression of fibrosis‐related genes in NRK‐49F cells and inflammation‐related genes in RAW264.7 cells. In NRK‐49F cells, IL‐6 and TGF‐β 1 treatment for 1h increased the expression of various fibrosis‐related genes (Figure 4c), especially Snai1 (Grande et al., 2015; Simon‐Tillaux & Hertig, 2017), Serpine1 (Flevaris & Vaughan, 2017; Ghosh & Vaughan, 2012), and Cxcr4 (Tang et al., 2019; Yuan et al., 2015), which are reported to be associated with kidney fibrosis. We also focused on Ccl19 (Sato & Yanagita, 2017) and Ccr1 (Eis, 2004; Vielhauer et al., 2004) in IL‐6‐treated RAW264.7 cells because they are suggested to be related to kidney fibrosis (Figure 4d). In the following experiments, we examined the involvement of these five genes (Snai1, Serpine1, Cxcr4, Ccl19, and Ccr1) in the lowering effects of STX‐0119 on kidney fibrotic genes.

### 3.5 STX‐0119 administration downregulates Cxcr4 and Ccr1 expression in UUO kidneys

**Figure 5:**
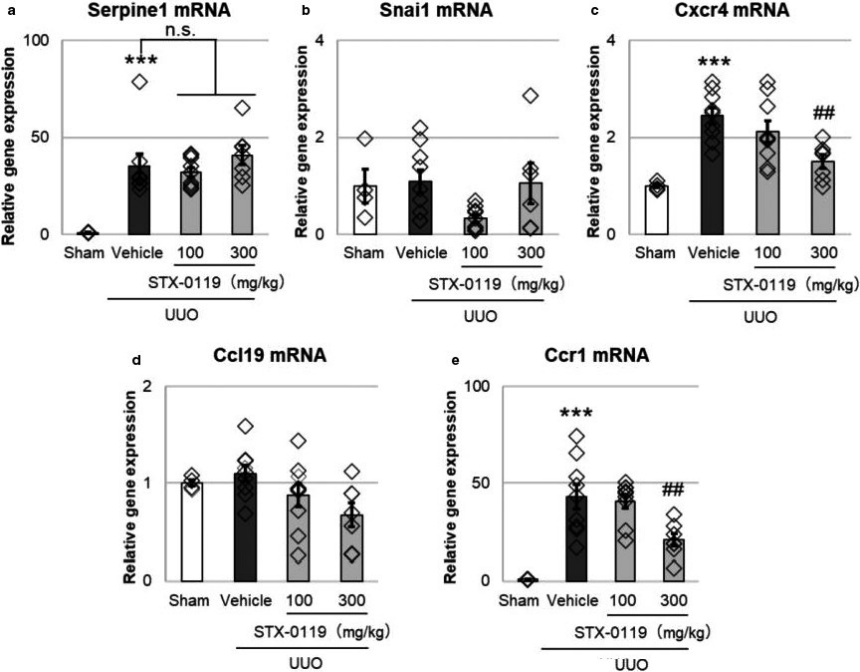
Effects of STX‐0119 on fibrosis‐related genes identified in vitro. (a‐e) The mice were sacrificed at day 3 after UUO surgery, and mRNA was extracted and reverse transcribed. Gene expression was detected by qPCR. Each gene was normalized by Gapdh mRNA. ***p < .001 versus sham, ##*p* < .01 versus UUO + Vehicle; one‐way analysis of variance + Tukey's test. n.s., not significant. *N* = 4 for sham group and *N* = 9 for other groups

We examined the effects of STX‐0119 on the five genes mentioned above in obstructed kidneys. Serpine1, Cxcr4, and Ccr1 expression was increased by UUO surgery, but there was no change in the expression of Ccl19 and Snai1. In addition, STX‐0119 administration did not affect Serpine1 expression, whereas Cxcr4 and Ccr1 levels were significantly decreased by STX‐0119 (Figure 5a–e). Antagonism of Cxcr4 delays the progression of kidney fibrosis in UUO kidneys (Tang et al., 2019) and Ccr1 knockout mice are less susceptible to kidney fibrosis (Eis, 2004). These results suggest that Cxcr4 and Ccr1 play a role in the antifibrotic effects of STX‐0119.

### 3.6 STX‐0119 administration decreases Cxcr4 expression without affecting STAT3 phosphorylation in kidney fibroblasts

**Figure 6:**
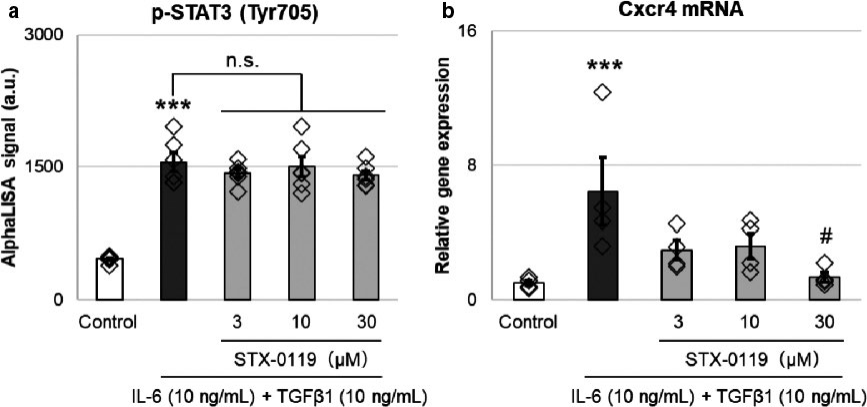
Effects of STX‐0119 on the expression of p‐STAT3 protein (a) and Cxcr4 mRNA (b) in NRK‐49F cells. (a) NRK‐49F cells were treated with STX‐0119 for 30 min, following 10‐min treatment with IL‐6 and TGF‐β1. Then, the expression of p‐STAT3 in cell lysates was determined by p‐STAT3 AlphaLISA. *N* = 5. (b) NRK‐49F cells were treated with STX‐0119 for 30 min, following 1‐hr treatment with IL‐6 and TGF‐β1. mRNA was extracted and Cxcr4 gene expression was detected by qPCR. Cxcr4 gene expression was normalized by Gapdh mRNA. *N* = 5. **p < .01, ***p < .001 versus sham; #*p* < .05 versus UUO + Vehicle; one‐way analysis of variance + Tukey's test. n.s., not significant

Cxcr4 is expressed in tubules; therefore, we investigated the effect of STX‐0119 on Cxcr4 expression using NRK‐49F cells. The increase in phosphorylated STAT3 levels by IL‐6 and TGF‐β 1 treatment were not changed by STX‐0119 (Figure 6a). Irrespective of STAT3 phosphorylation, Cxcr4 gene expression was significantly diminished by pretreatment with STX‐0119 (Figure 6b). We consider that STX‐0119 has an inhibitory effect on Cxcr4 gene expression without affecting STAT3 phosphorylation in kidney fibroblasts.

### 3.7 STX‐0119 administration downregulates Ccr1 expression in parallel with STAT3 phosphorylation in blood cells from UUO mice

**Figure 7:**
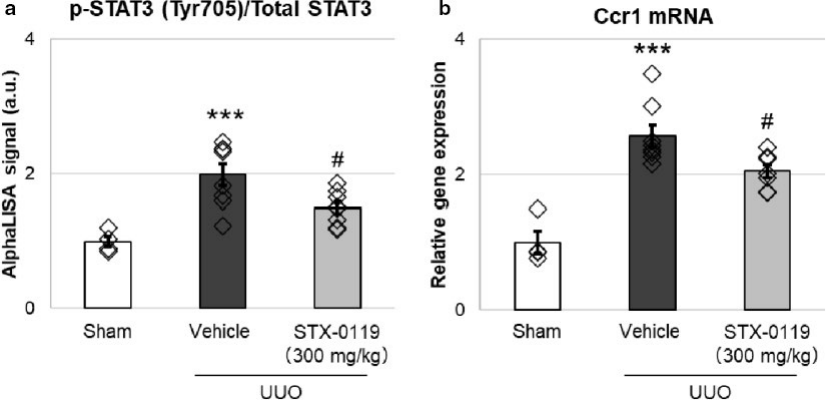
Effects of STX‐0119 on the expression of p‐STAT3/STAT3 protein (a) and Ccr1 mRNA (b) in blood cells from UUO mice. (a) Blood cells were collected from mouse blood at day 1 after UUO surgery. STX‐0119 was administered just before UUO surgery. Expression of p‐STAT3 and total STAT3 protein in blood cells was determined by AlphaLISA. (b) mRNA was extracted from blood cells collected from mouse blood at day 1 after UUO surgery. Ccr1 gene expression was detected by qPCR. Ccr1 gene expression was normalized by Gapdh mRNA. **p < .01, ***p < .001 versus sham; #*p* < .05 versus UUO + Vehicle; one‐way analysis of variance + Tukey's test. *N* = 4 for sham group and *N* = 8 for other groups

Ccr1 is expressed mainly in leukocytes and is suggested to be involved in the migration of leukocytes into inflamed tissues. We examined the change in Ccr1 expression and STAT3 phosphorylation in blood cells. STAT3 phosphorylation was significantly increased in blood cells and this was reversed by STX‐0119 administration (Figure 7a). In parallel with this, Ccr1 gene expression was decreased in blood cells from UUO mice (Figure 7b). These results suggest that STX‐0119 inhibits STAT3 activation by regulating its phosphorylation state and this leads to the downregulation of Ccr1 expression.

## 4 DISCUSSION

In this study, we have demonstrated the preventive effects of STX‐0119 on fibrosis markers using a mouse model of kidney fibrosis. This is the first report to indicate that STX‐0119 inhibits fibrotic gene expressions in the kidney and to elucidate a part of the underlying mechanism.

First, we investigated whether STAT3 activation was induced in mouse UUO kidneys. IL‐6 expression, STAT3 phosphorylation and STAT3 target gene expression were increased in obstructed kidneys. These results indicate that STAT3 is activated in kidneys by UUO. Arakawa et al. reported that p‐STAT3‐positive tubulointerstitial cells are significantly increased in the kidneys of IgA nephropathy patients compared with normal kidneys (Arakawa et al., 2008). Thus, it can be speculated that STAT3 is activated in human fibrotic kidneys.

STX‐0119 is an inhibitor of STAT3 dimerization and is reported to have antifibrotic effects on a liver fibrosis model (Choi et al., 2019). Therefore, in this study, we expected that STX‐0119 would exert antifibrotic effects in a mouse model of kidney fibrosis. As expected, STX‐0119 suppressed the expression of fibrosis‐related genes in obstructed kidneys. Pang et al. suggested that S3I‐201, an inhibitor of STAT3 phosphorylation, inhibits kidney fibrosis in parallel to suppressing STAT3 phosphorylation in UUO kidneys (Pang et al., 2010). On the other hand, we demonstrated that STX‐0119 inhibited the expression of STAT3 target genes without affecting STAT3 phosphorylation. These results suggest that STX‐0119 exerts its antifibrotic effects by a mechanism different from that of S3I‐201.

In this study, we focused on kidney fibroblasts and leukocytes to clarify the mechanism of STX‐0119. We first examined the effect of STX‐0119 on the leukocyte marker expressions in UUO kidneys. STX‐0119 decreased leukocyte marker expression in obstructed kidneys. From these results, we assumed that the effects of STX‐0119 are partly mediated by the inhibition of leukocyte infiltration.

Next, we investigated which genes were upregulated by STAT3 activation in kidney fibroblasts and leukocytes in vitro. Various genes that reported to be involved in kidney fibrosis were upregulated by STAT3 activation, that is, IL‐6 and TGF‐β 1 treatment. From these genes, we selected Snai1, Serpine1, and Cxcr4 for kidney fibroblasts, and Ccl19 and Ccr1 for leukocytes as candidate targets of the antifibrotic effects of STX‐0119 because they are upregulated by STAT3 activation in vitro. In particular, the expression of Cxcr4 and Ccr1 was downregulated in UUO kidneys by STX‐0119, and so we focused on these two genes in the following experiments.

Cxcr4 is ubiquitously expressed and has a single known ligand, stromal cell‐derived factor‐1α (Tachibana et al., 1998). Yuan et al. showed that the CXCR4 antagonist provides protection from UUO‐induced fibrosis (Yuan et al., 2015). In this study, we found that Cxcr4 was upregulated by UUO kidneys and this upregulation was suppressed by STX‐0119. For in vitro experiments, we used NRK‐49F cells, and STX‐0119 inhibited the upregulation of Cxcr4 expression induced by IL‐6 and TGF‐β 1 treatment. Thus, we suggest that the preventive effects of STX‐0119 on kidney fibrotic genes are partially mediated by Cxcr4 downregulation in kidney fibroblasts. Recently, Liu et al. reported the upregulation of Cxcr4 in tubular cells in kidneys from patients with IgA nephropathy, rapidly progressing glomerulonephritis, and FSGS (Liu et al., 2020). Therefore, Cxcr4 blockade may be effective not only in animal models but also in human fibrotic kidneys.

Ccr1 expresses mainly in leukocytes such as macrophages and T cells. Previous studies have demonstrated that Ccr1 mRNA expression is increased in fibrotic kidneys (Vielhauer et al., 2001). Analysis of UUO kidneys from Ccr1‐deficient mice revealed a reduction of renal fibrosis and interstitial lymphocytes compared with wild‐type controls (Eis, 2004). In this study, we demonstrated that Ccr1 expression in blood cells was upregulated in UUO mice, and that STX‐0119 administration significantly decreased Ccr1 expression in blood cells. Ccr1 expression was markedly increased by IL‐6 treatment in RAW264.7 cells. Additionally, Shin et al. showed that STAT3 is necessary for CCR1 promoter activation in breast cancer cells (Shin et al., 2017). Collectively, these data suggest that Ccr1 in leukocytes is induced by STAT3 activation and that STX‐0119 decreases Ccr1 expression, which contributes to the reduction of leukocyte infiltration into UUO mouse kidneys. Recently, many researchers have reported the involvement of Ccr1 in the pathogenesis of CKD models (Anders et al., 2006). Therefore, we can also expect that a STAT3 inhibitor would improve the pathophysiology of CKD models.

STX‐0119 is reported to be an inhibitor of STAT3 dimerization; however, we think that STX‐0119 can also inhibit STAT3 phosphorylation in certain cell types because STX‐0119 binds to the SH2 domain, which is a phosphorylation site of STAT3 (Matsuno et al., 2010). We showed that STAT3 phosphorylation was not affected in UUO kidneys, but was decreased in blood cells of UUO mice by STX‐0119 administration. We cannot presently explain why these phenomena occurs, but they may be derived from differences in the STAT3 phosphorylation mechanisms between cell types, for instance, the expression of upstream kinases.

In summary, we have demonstrated the preventive effects of STX‐0119 on fibrotic gene upregulation in UUO kidneys. In addition, STX‐0119 decreased the expression of Cxcr4 in kidney cells and Ccr1 in blood cells. Thus, we assumed that STX‐0119 suppressed upregulation of kidney fibrosis markers via the downregulation of Cxcr4 in kidney fibroblasts and Ccr1 in leukocytes. However, our research has a limitation; we did not evaluate fibrosis by histological analysis and protein expression. These points should be addressed in the future study, and we believe STAT3 dimerization inhibitor exerts antifibrotic effects in fibrotic kidneys because STAT3 phosphorylation inhibitor is reported to have antifibrotic effect from histological point of view (Pang et al., 2010). After all, this is the first report to demonstrate the suppression of fibrosis‐related genes in kidneys by a STAT3 dimerization inhibitor, and to suggest that STX‐0119 may be a new candidate for treating CKD via the slowing of kidney fibrosis.

## CONFLICT OF INTEREST

The authors declare no conflict of interest in association with this study.

## AUTHORS' CONTRIBUTIONS

Makitani, Ogo, and Asai designed the study. All the experiments were performed by Makitani. All the authors discussed the results and implications and commented on the manuscript at all stages.

5

**Table 1:** List of TaqMan assay IDs used in this study

Gene	TaqMan assay ID
Mouse Il‐6	Mm00446190_m1
Mouse Myc	Mm00487804_m1
Mouse Ccl12	Mm01617100_m1
Mouse Cxcl10	Mm00445235_m1
Mouse Col1a1	Mm00801666_g1
Mouse Col3a1	Mm00802300_m1
Mouse Col4a1	Mm01210125_m1
Mouse Acta2	Mm00725412_s1
Mouse Tgfb1	Mm01178820_m1
Mouse Ptprc	Mm01293577_m1
Mouse Itgam	Mm00434455_m1
Mouse Serpine1	Mm00435858_m1
Mouse Snai1	Mm00441533_g1
Mouse Cxcr4	Mm01996749_s1
Mouse Ccl19	Mm00839966_g1
Mouse Ccr1	Mm00438260_s1
Mouse Gapdh	Mm99999915_g1
Rat Cxcr4	Rn01483207_m1
Rat Gapdh	Rn01775763_g1
